# Supplementary material for: A hybrid piezoelectric resonator-based DC-DC converter
Source: Nat Commun. 2026 Mar 17;17:4054. doi: 10.1038/s41467-026-70494-0 (PMC13139477; doi:10.1038/s41467-026-70494-0)
Supplement: Supplementary file 1 — Supplementary Information [file 41467_2026_70494_MOESM1_ESM.pdf]

Supplementary Information for
A Hybrid Piezoelectric Resonator-based DC-DC Converter

Jae-Young Ko[1], Wen-Chin B. Liu[1], Patrick P. Mercier[1]*

[1] Department of Electrical and Computer Engineering, University of California, San Diego, La Jolla, CA, 92093, USA

* E-mail: pmercier@ucsd.edu (P.-P. Mercier)

Supplementary Note1. CHARGE BALANCE AND ENERGY BALANCE OF PIEZOELECTRIC RESONATOR

In general, the piezoelectric resonator (PR) must satisfy charge balance and energy balance over an operation cycle. Fig.2a in the main text presents a representative waveform example operating over 7 phases, which is used here to demonstrate the application of charge and energy balance of the PR. Accordingly, the charge balance of PR mechanical capacitor, C, and the piezoelectric static capacitance, C_P, can be written in terms of q_n as follows

$$q_1 + q_2 + q_3 + q_4 + q_5 + q_6 + q_7 = 0 \qquad (S1)$$

$$q_1 + q_3 + q_5 + q_7 = 0 \qquad (S2)$$

During the connected states of the PR, corresponding to ø2, ø4, and ø6, the PR stores, releases, or redistributes energy, denoted as E_2, E_4, and E_6, respectively. The voltage across the PR terminals in these phases are defined as V_2, V_4, and V_6. Under these definitions, the energy balance of the PR can be written as follows.

$$E_2 + E_4 + E_4 = V_2 q_2 + V_4 q_4 + V_6 q_6 = 0 \qquad (S3)$$

Supplementary Note 2. DERIVATION OF UTILIZATION FACTOR (*K*) AND CURRENT AMPLITUDE OF PIEZOELETRIC RESONATOR ($I_{L,PK}$)

Here, we derive the expressions for the utilization factor *K* and the current amplitude of piezoelectric resonator(PR) referenced in the main text. Based on the charge and energy balance of PR described in Supplementary Note1, the voltage conversion ratio (V_{IN}/V_{OUT}) and utilization factor *K* of this work is expressed as

$$\frac{V_{OUT}}{V_{IN}} = \frac{q_2}{9q_2 + 3q_4} \le \frac{1}{9} \qquad (S4)$$

$$K = \frac{|q_2| + |q_6|}{|q_2| + |q_4| + |q_6|} = \frac{2|q_2| + |q_4|}{2|q_2| + 2|q_4|} = \frac{V_{IN} - 3V_{OUT}}{2(V_{IN} - 6V_{OUT})} \qquad (S5)$$

Due to the resonant operation, the PR current $i_{L,PR}$ can be approximated as a pure sinusoidal waveform with frequency $w = 2\pi f_{sw}$. As illustrated in Supplementary Fig.3, with amplitude $I_{L,PK}$, the PR current is expressed as

$$i_{L,PR}(t) = I_{L,PK} \sin wt \qquad (S6)$$

The PR's total charge Q_{total} comprises the charge used for energy delivery and the charge required to achieve zero-voltage switching, $C_P V_{PP}$, where V_{PP} denotes the voltage swing of the PR. Therefore, the total charge can be written as

$$Q_{total} = \int \left|I_{L,PR}(t)\right| dt = |q_2| + |q_4| + |q_6| + 2C_P(V_{IN} - 3V_{OUT}) \tag{S7}$$

In our hybrid PR DC-DC converter, the energy-storing and -delivering elements consist of the PR(Q_{PR}) and the capacitors(Q_{CFs}). From Supplementary Fig.3, the energy delivered to the output and the energy received from the input can be express as

$$Q_{OUT} = Q_{PR} + Q_{CFs} = 6|q_2| + 3|q_6| = \frac{P_{OUT}}{f_{sw}V_{OUT}} \tag{S8}$$

$$Q_{IN} = |q_2| = \frac{P_{IN}}{f_{sw}V_{IN}} \tag{S9}$$

From Eqs. (S8), (S9) and definition of K, the energy delivered by the PR (Q_{PR}) to the output and the total charge Q_{total} are derived as follows

$$Q_{PR} = |q_2| + |q_6| = \frac{Q_{OUT} - 3|q_2|}{3} \tag{S10}$$

$$Q_{total} = \frac{Q_{PR}}{K} + 2C_P(V_{IN} - 3V_{OUT}) = \frac{1}{3K}\left(\frac{P_{OUT}}{f_{sw}V_{OUT}} - \frac{3P_{IN}}{f_{sw}V_{IN}}\right) + 2C_P(V_{IN} - 3V_{OUT}) \tag{S11}$$

From Eqs. (S6) and (S7), the current amplitude of PR, $I_{L,PK}$, is obtained as follows

$$I_{L,PK} = \frac{\pi}{2} f_{sw} Q_{total} = \frac{\pi}{2}\left[\frac{1}{3K}\left(\frac{P_{OUT}}{V_{OUT}} - \frac{3P_{IN}}{V_{IN}}\right) + 2f_{sw}C_P(V_{IN} - 3V_{OUT})\right] \tag{S12}$$

Supplementary Note 3. CHARGE-BASED OUTPUT VOLTAGE RIPPLE ANALYSIS

We investigate the reduction of the output voltage ripple using a charge-based analysis. As observed in Fig.3c of the main text, the output voltage ripple is affected by the ratio of energy delivered to the output during positive (Q_p) and negative (Q_n) PR current intervals. This energy ratio corresponds directly to the charge ratio. From Eqs. (S1), (S2), and (S4), the ratio of q_6 to q_2 is given by

$$\frac{|q_6|}{|q_2|} = \frac{V_{IN} - 6V_{OUT}}{3V_{OUT}} = \frac{VCR}{3} - 2 \tag{S13}$$

Here, VCR denotes the voltage conversion ratio (V_{IN}/V_{OUT}). Using Eq. (S13) and Supplementary Fig.3, the ratio of Q_p to Q_n can be expressed as follow

$$Q_p = 3|q_2| + |q_6| \tag{S14}$$

$$Q_n = 3|q_2| + 2|q_6| \tag{S15}$$

$$Q_p : Q_n = \left(\frac{VCR}{3} + 1\right) : \left(\frac{2}{3}VCR - 1\right) \tag{S16}$$

For comparison, the DSPPR (reference [34]) operating in a partial dual-path configuration is analyzed using the same approach. The charge distribution is shown in Supplementary Fig.4a from which the following results are obtained as

$$q_{2,DSPPR}\left(\frac{V_{IN}}{2} - 2V_{OUT}\right) + q_{6,DSPPR}(2V_{OUT}) = 0 \tag{S17}$$

$$\frac{\left|q_{6,DSPPR}\right|}{\left|q_{2,DSPPR}\right|} = \frac{V_{IN} - 4V_{OUT}}{4V_{OUT}} = \frac{VCR}{4} - 1 \tag{S18}$$

$$Q_{p,DSPPR} = \left|q_{2,DSPPR}\right| \tag{S19}$$

$$Q_{n,DSPPR} = |q_{2,DSPPR}| + 2|q_{6,DSPPR}| \quad (S20)$$

$$Q_{p,DSPPR}: Q_{n,DSPPR} = 1: \left(\frac{VCR}{2} - 1\right) \quad (S21)$$

Supplementary Fig.4b shows Eqs. (16) and (21) plotted as a function of VCR, confirming that the proposed converter achieves effective current distribution even at high VCR. Supplementary Fig.4c presents the output waveforms and corresponding ripples for each topology under identical condition, further validating the analysis.

Supplementary Note 4. SWITCHING TIMING ANALYSIS

Supplementary Fig.7 illustrates the turn-on and turn-off events of all switches in each phase over time and includes a brief description. Using this information and Eqs. (S6) and (S12), the switching timings of the individual switches can be calculated.

The beginning of the cycle is defined as t=0. Switches S1 turns on at t=0 and starts to operate in the open state. During the open state, the PR current charges or discharges C_P, allowing the corresponding charge to be calculated from the voltage variation across C_P (ΔV_{CP}).

$$q_{open} = C_P \Delta V_{CP} = \int I_{L,PK} \sin wt \, dt \quad (S22)$$

From this, the turn-on timings of S7 (t_0) and S3 (t_1) are expressed as

$$t_0 = \frac{1}{w} \cos^{-1}\left(1 - \frac{w}{I_{L,PK}} 2C_P V_{OUT}\right) \quad (S23)$$

$$t_1 = \frac{1}{w} \cos^{-1}\left(1 - \frac{w}{I_{L,PK}} 3C_P V_{OUT}\right) \quad (S24)$$

Next, since q_2 is known from Eq. (S9), the S1 turn-off timing at the end of ø2 (t_2) is calculated as follows

$$t_2 = \frac{1}{w} \cos^{-1}\left[1 - \frac{w}{I_{L,PK}}\left(\frac{P_{IN}}{f_{sw} V_{IN}} + 3C_P V_{OUT}\right)\right] = \frac{1}{w} \cos^{-1}\left[1 - \left(\frac{2\pi P_{OUT}}{I_{L,PK} V_{IN}} + \frac{3wC_P V_{OUT}}{I_{L,PK}}\right)\right] \quad (S25)$$

Similarly, ø3 is open state, and the end of ø3 (S2 turn-on timing, t_3) is derived as

$$t_3 = \frac{1}{w} \cos^{-1}\left[1 - \left\{\frac{2\pi P_{OUT}}{I_{L,PK} V_{IN}} + \frac{wC_P (V_{IN} - 3V_{OUT})}{I_{L,PK}}\right\}\right] \quad (S26)$$

S3 turns off when the PR current becomes negative. Since this occurs at half of the cycle, the turn-off timing of S3 (t_4) is shown below

$$t_4 = \frac{1}{2f_{sw}} \quad (S27)$$

Since ø5, like ø1, is an open state starting with the PR current at zero, the turn-on timing of S9 (t_5) and S4 (t_6) are calculated as follows

$$t_5 = \frac{1}{2f_{sw}} + \frac{1}{w} \cos^{-1}\left[1 - \frac{w}{I_{L,PK}} C_P V_{OUT}\right] \quad (S28)$$

$$t_6 = \frac{1}{2f_{sw}} + t_1 \quad (S29)$$

The turn-off timing of S2 (t_7) is set to ensure that V_{X1} reaches V_{IN} at the end of ø7

$$t_7 = \frac{1}{f_{sw}} - \frac{1}{w}\cos^{-1}\left[1 - \frac{w}{I_{L,PK}} C_P (V_{IN} - 6V_{OUT})\right] \quad (S30)$$

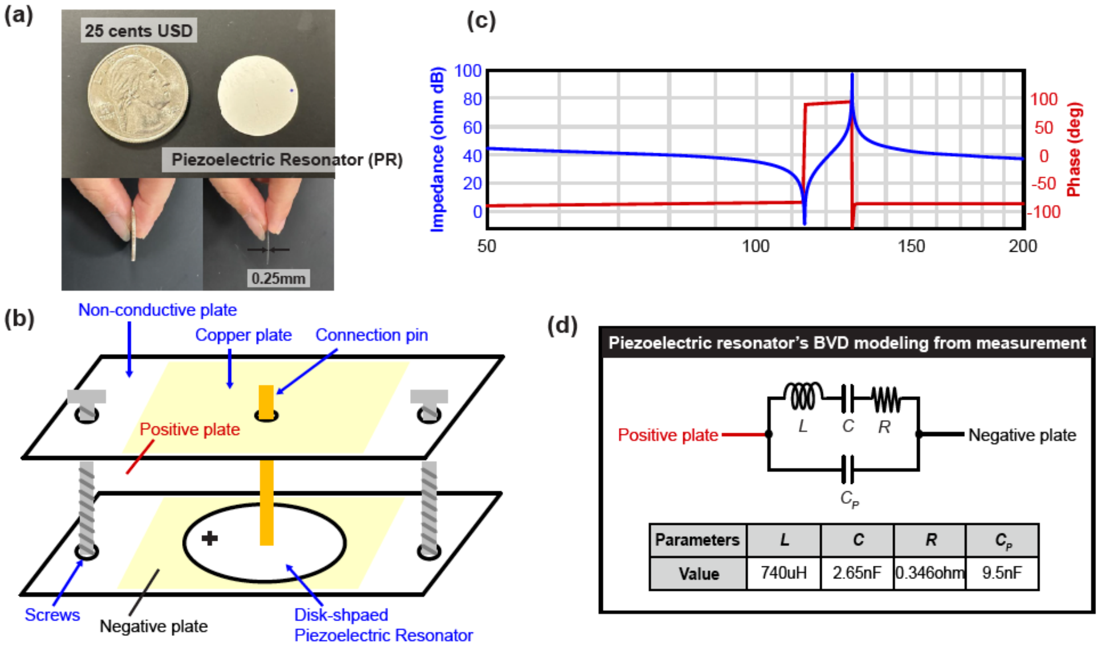

Supplementary Figure 1. PR mounting strategy and calculated BVD modeling parameters from measurements. a, Planar form-factor of the PR compared to a 25 cent USD coin. **b**, Mounting strategy in which the PR is placed between the upper plate connected to the positive port and the bottom plate connected to the negative port, with the PR allowed to vibrate using gold pins and screws to connect it to both plates. **c**, Frequency response of the tested PR, as shown in (a), with mounting strategy like (b), showing the inductive operation region from 115kHz to 129kHz. **d**, Calculated BVD modeling value from the frequency response in (c).

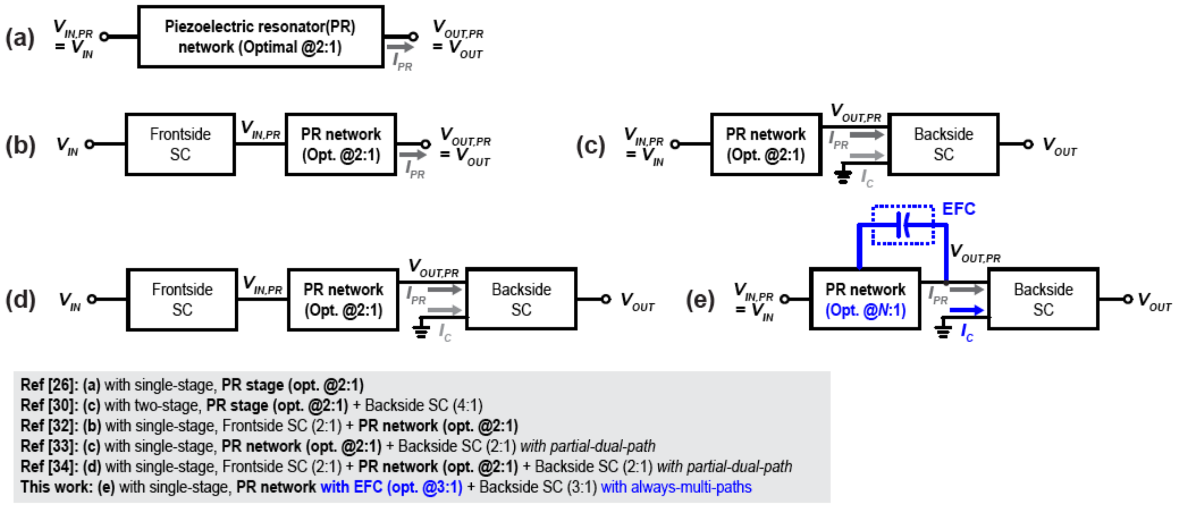

Supplementary Figure 2. Generalized the state-of-the-art PR-based topologies. a, Pure PR-based DC-DC converter. **b**, PR-based DC-DC converter with frontside SC. **c**, PR-based DC-DC converter with backside SC. **d**, PR-based DC-DC converter with front/backside SC. **e,** Generalized concept of the proposed AMP-EFC converter.

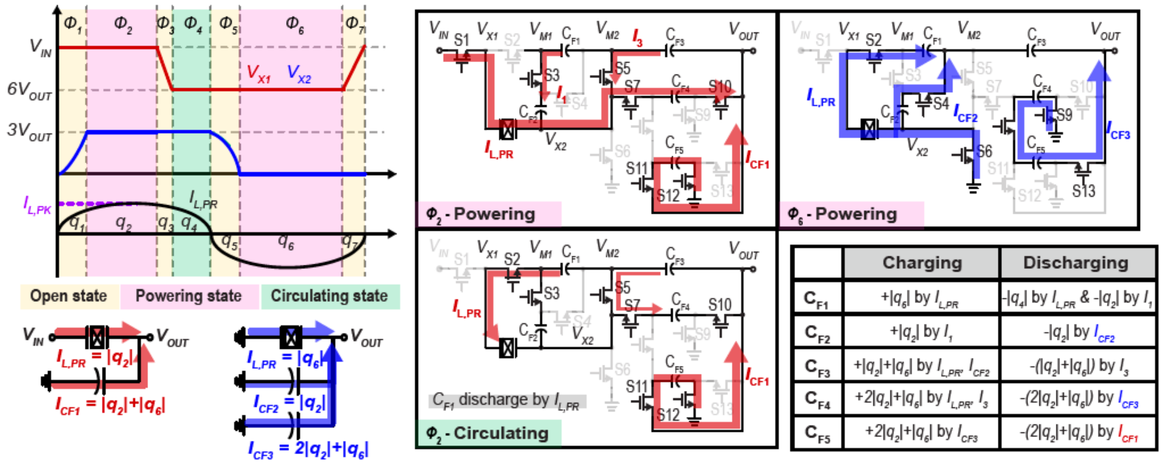

Supplementary Figure 3. Charging/discharging charge of each flying capacitor considering multiple current paths ($I_{L,PR}$, I_{CF1}-I_{CF3})

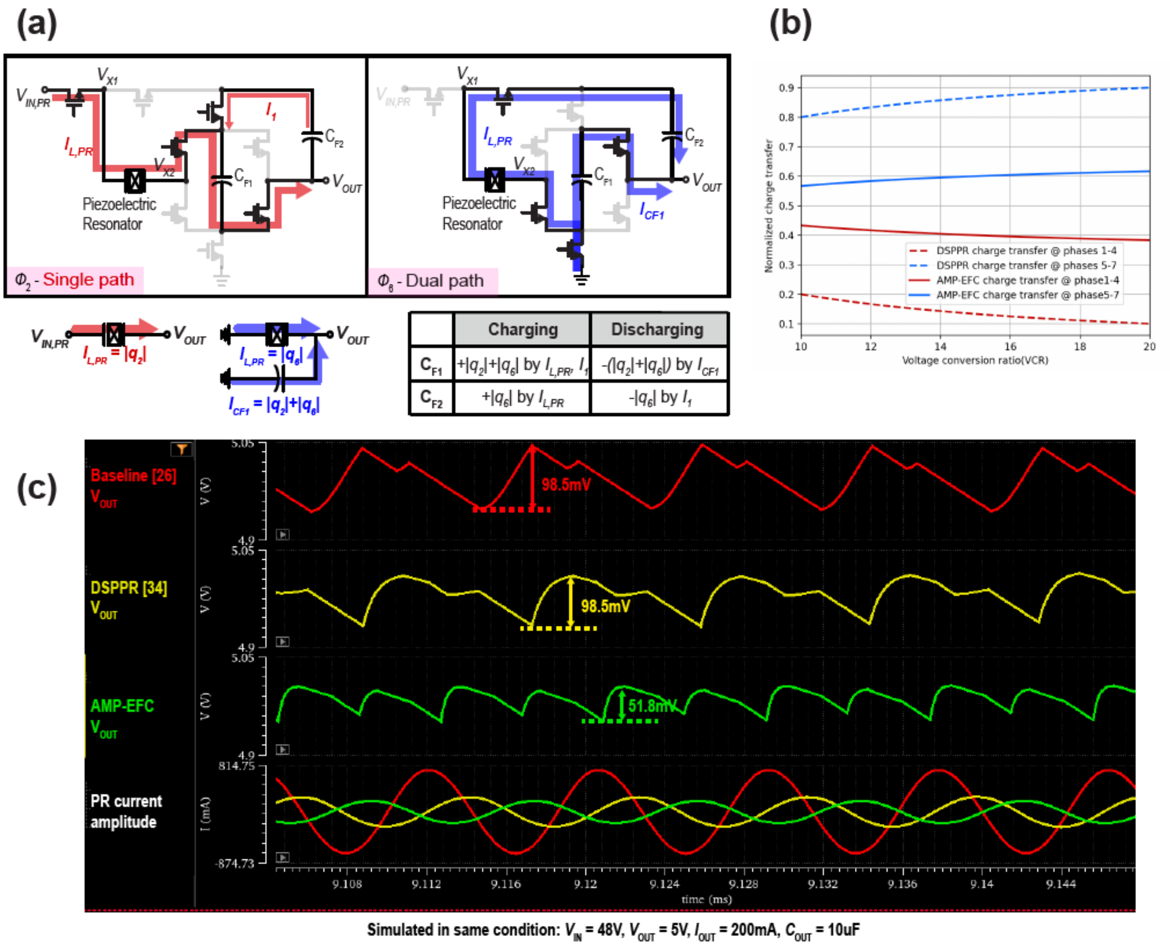

Supplementary Figure 4. Analysis and comparison of output voltage ripple. a, Charge-based analysis of partial dual-path PR-based converter [34]. **b**, Comparison of transferred charge per half-cycle between DSPPR [34] and the proposed AMP-EFC. **c**, Simulated voltage ripple and PR current at V_{IN}=48V, V_{OUT}=5V, I_{OUT}=200mA, and C_{OUT}=10uF.

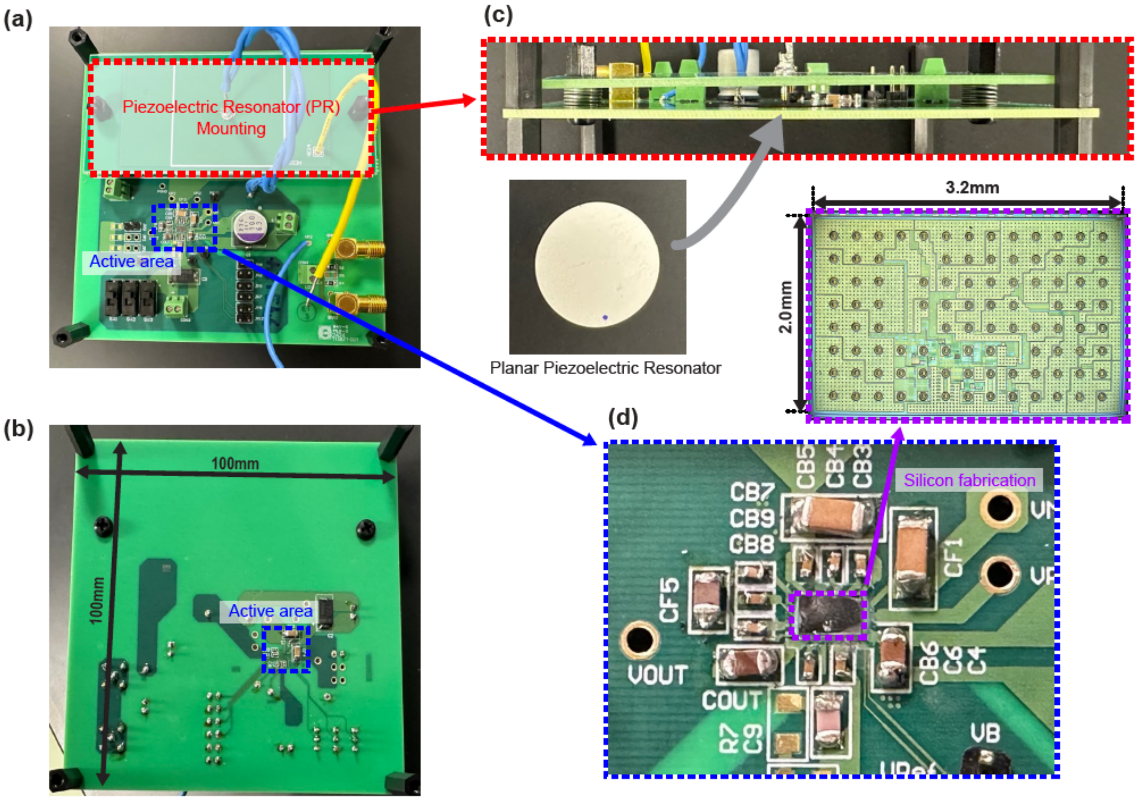

Supplementary Figure 5. Printed circuit board (PCB) with PR mounting for testing. a, Top view of PCB with assembly. **b**, Bottom view of PCB with assembly. **c**, Planar PR mounting part. **d**, Zoom in the active area on the top of PCB.

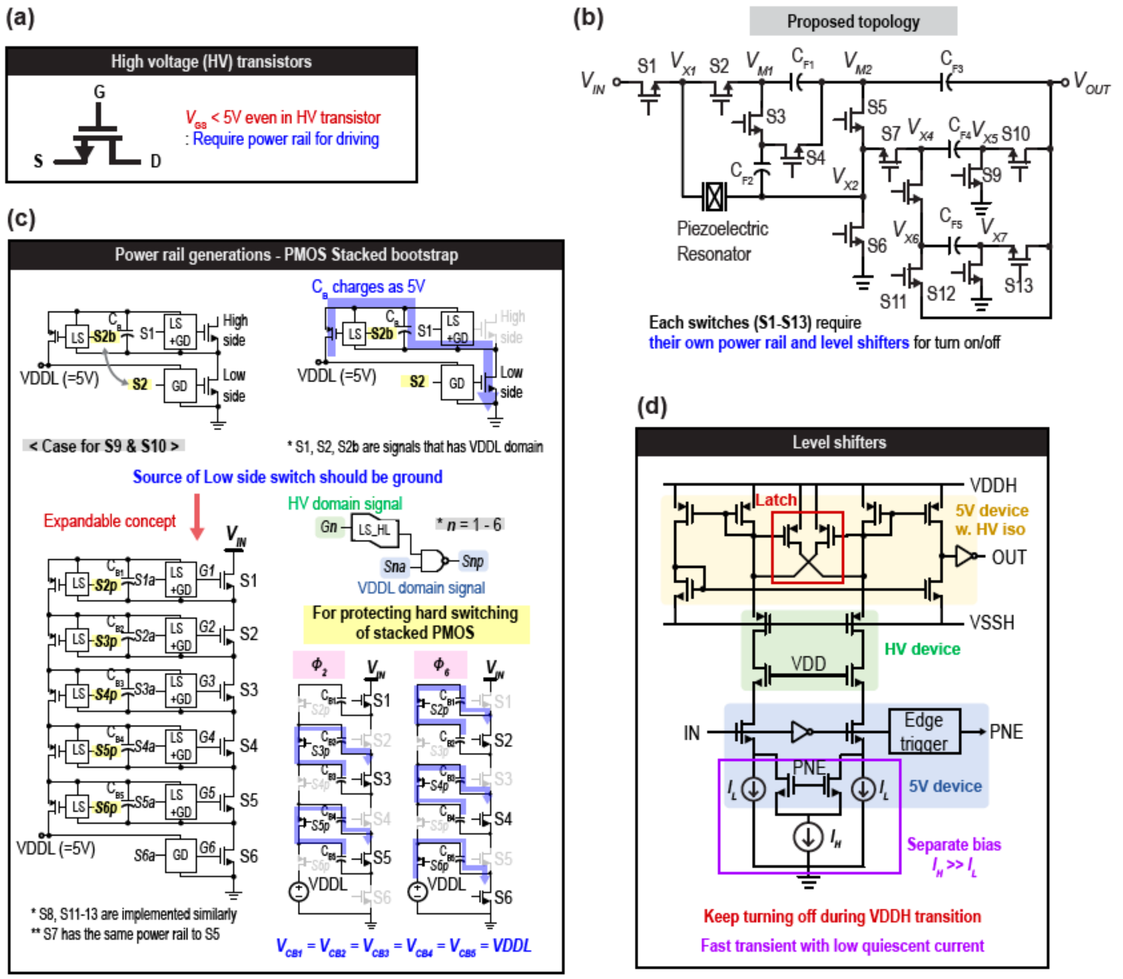

Supplementary Figure 6. Detailed driving rail generation and level shifter circuits. a, Requirement of the driving voltage (V_{GS}) for HV switches, which remains below 5V, even if the V_{DS} can withstand >10V. **b**, Requirement of driving circuits in the reported AMP-EFC PR-based converter. **c**, Concept of power rail generation circuits and implementations. **d**, A level shifter that quickly and reliably raises the driving signals to the target power rail level with low quiescent current.

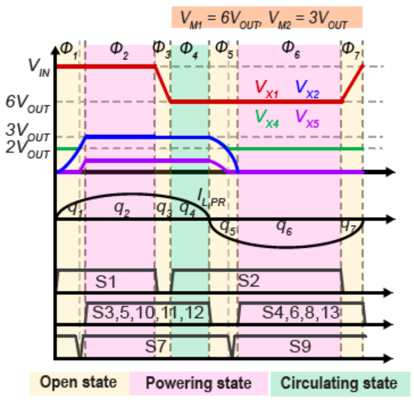

	Turn-on timing	Turn-off timing
S1	Begining of the cycle (t=0)	Voltage regulation: $\lvert q_2\rvert = P_{IN} / f_{sw} V_{IN} = P_{OUT} / f_{sw} V_{IN}$
S2	Zero voltage switching at $V_{X1} = V_{M1}$ (=$6V_{OUT}$)	Ensure V_{X1} reach to V_{IN} at the end of the $\Phi 7$
S3	Zero voltage switching at $V_{X2} = V_{M2}$ (=$3V_{OUT}$)	Half cycle ($t=T_s/2$)
S4	Zero voltage switching at $V_{X2} = 0$	End of the cycle ($t=T_s$)
S7	Zero voltage switching at $V_{X2} = V_{X4}$	Zero voltage switching at $V_{X5} = 0$
S9	Zero voltage switching at $V_{X5} = 0$	Zero voltage switching at $V_{X2} = V_{X4}$

* S5, 10, 11, 12 has same timing with S3
** S6, 8, 13 has the same timing with S4
*** There is dead-time between S7 and S9 signals

Supplementary Figure 7. Table summarizing the on/off timing strategy and the corresponding timing diagram.
